# Nicotine-Induced Effects on Nicotinic Acetylcholine Receptors (nAChRs), Ca^2+^ and Brain-Derived Neurotrophic Factor (BDNF) in STC-1 Cells

**DOI:** 10.1371/journal.pone.0166565

**Published:** 2016-11-15

**Authors:** Jie Qian, Shobha K. Mummalaneni, Reem M. Alkahtani, Sunila Mahavadi, Karnam S. Murthy, John R. Grider, Vijay Lyall

**Affiliations:** Department of Physiology and Biophysics, Virginia Commonwealth University, Richmond, Virginia, 23219, United States of America; The University of Tokyo, JAPAN

## Abstract

In addition to the T2R bitter taste receptors, neuronal nicotinic acetylcholine receptors (nAChRs) have recently been shown to be involved in the bitter taste transduction of nicotine, acetylcholine and ethanol. However, at present it is not clear if nAChRs are expressed in enteroendocrine cells other than beta cells of the pancreas and enterochromaffin cells, and if they play a role in the synthesis and release of neurohumoral peptides. Accordingly, we investigated the expression and functional role of nAChRs in enteroendocrine STC-1 cells. Our studies using RT-PCR, qRT-PCR, immunohistochemical and Western blotting techniques demonstrate that STC-1 cells express several α and β nAChR subunits. Exposing STC-1 cells to nicotine acutely (24h) or chronically (4 days) induced a differential increase in the expression of nAChR subunit mRNA and protein in a dose- and time-dependent fashion. Mecamylamine, a non-selective antagonist of nAChRs, inhibited the nicotine-induced increase in mRNA expression of nAChRs. Exposing STC-1 cells to nicotine increased intracellular Ca^2+^ in a dose-dependent manner that was inhibited in the presence of mecamylamine or dihydro-β-erythroidine, a α4β2 nAChR antagonist. Brain-derived neurotrophic factor (BDNF) mRNA and protein were detected in STC-1 cells using RT-PCR, specific BDNF antibody, and enzyme-linked immunosorbent assay. Acute nicotine exposure (30 min) decreased the cellular content of BDNF in STC-1 cells. The nicotine-induced decrease in BDNF was inhibited in the presence of mecamylamine. We also detected α3 and β4 mRNA in intestinal mucosal cells and α3 protein expression in intestinal enteroendocrine cells. We conclude that STC-1 cells and intestinal enteroendocrine cells express nAChRs. In STC-1 cells nAChR expression is modulated by exposure to nicotine in a dose- and time-dependent manner. Nicotine interacts with nAChRs and inhibits BDNF expression in STC-1 cells.

## Introduction

Our sense of taste helps us to determine whether the food is nutritious and should be ingested or is potentially toxic and should be rejected [1]. Taste also contributes to palatability [2], satiation, thermogenic effects [3] and the reward value of food [4]. A distinct subset of taste receptor cells (TRCs) in the taste buds on the tongue detect taste stimuli representing the five primary taste qualities salty, sour, sweet, bitter, and umami [5]. Bitter, sweet and umami taste is detected by TRCs that express G-protein coupled taste receptors (GPCRs; T1Rs and T2Rs), PLC**β**2 and TRPM5. Salty taste is perceived by TRCs that express the amiloride- and Bz-sensitive epithelial Na^+^ channel (ENaC) [6–8]. Sour taste is perceived by TRCs that express PKD2L1 channels, carbonic anhydrase-4 [9, 10] and Zn^2+^-sensitive proton channels [8, 11, 12].

Likewise, enteroendocrine cells in the gut detect nutrients that we ingest via similar taste receptors and chemosensory signaling pathways [13–27]. The ingested nutrients in the gut lumen regulate the release of gastrointestinal hormones and neurohumoral peptides that play a role in gut secretion and motility as well as in controlling appetite and satiety by activating the gut-brain axis [22, 23, 27].

In addition to the above classical taste receptors, nicotinic acetylcholine receptors (nAChRs) expressed in central and peripheral organs are emerging as major players in the regulation of appetite and body weight [23]. In line with this emerging role of nAChRs, we have recently shown that nAChRs serve as additional bitter taste receptors for nicotine, acetylcholine and alcohol [28]. Compared with wild-type (WT) mice, TRPM5 knockout (KO) mice have reduced, but not abolished, chorda tympani (CT) taste nerve responses to nicotine. In both genotypes, lingual application of mecamylamine (Mec), a non-specific nAChR-antagonist, inhibited neural and aversive behavioral responses to nicotine [29]. In addition to nicotine, CT responses to acetylcholine and ethanol were blocked by the nAChR modulators: Mec, dihydro-β-erythroidine (DHβE), and CP-601932 (a partial agonist of α3β4* nAChR). These studies suggest that neural and behavioral responses to nicotine are dependent upon two parallel bitter taste receptor-mediated pathways, a TRPM5-dependent pathway and a TRPM5-independent pathway. The first pathway is common to many other bitter tastants [29]. The second pathway is important not only for the detection of nicotine but is also involved in the detection of the bitter stimuli acetylcholine and ethanol, and is dependent upon the presence of nAChRs expressed in a subset of TRCs [28].

However, at present it is not clear if nAChRs are expressed in enteroendocrine cells other than beta cells of the pancreas [30] and enterochromaffin cells [31], and if they play a role in the synthesis and release of neurohumoral peptides. Accordingly, in this study, we investigated the expression and functional role of nAChRs in enteroendocrine STC-1 cells. STC-1 cell line is an established cell line of enteroendocrine cells of mouse small intestine and have been shown to express sweet, umami and bitter taste receptors and their downstream intracellular signaling effectors [14–16; 24, 25, 32]. Consistent with this, quinine, HCl, acetic acid, sucrose, sucralose, erythritol, NaCl, and monosodium glutamate induce cholecystokinin (CCK) release from STC-1 cells both in a concentration- and time-dependent manner [22, 25, 33]. STC-1 cells and mouse proximal small intestinal tissue explants also secreted CCK and inosine nucleotide (IMP) enhanced L-phenylalanine, L-leucine, and L-glutamate-induced, but not L-tryptophane-induced, CCK secretion [24]. These results suggest that activation of specific taste receptors leads to the release of specific hormones and effectors from STC-1 cells.

We investigated the expression of nAChRs in STC-1 cells using RT-PCR, real time (q)-RT-PCR, immunohistochemical and Western blot techniques. The functional role of nAChRs was investigated using nicotine as a bitter stimulus and Mec as a broad spectrum nAChR antagonists, on brain-derived neurotrophic factor (BDNF) content in STC-1 cells. BDNF is present in the gut and participates in survival and growth of enteric neurons, augmentation of enteric circuits, and stimulation of intestinal peristalsis and propulsion [34, 35]. To investigate if nAChRs are also expressed in intestinal tissue, we further tested for the presence of α3 and β4 mRNA in mouse intestinal mucosal cells and α3 expression in intestinal enteroendocrine cells using α3 antibody. In the taste system, BDNF and its cognate receptor trkB are present mainly in type III taste cells [36, 37] and are required for the developmental remodeling of taste bud innervation [38] and to maintain normal amounts of innervation to adult taste buds [39–41]. Exposure of neonate rats to nicotine causes a decrease in the expression of nerve growth factor and BDNF and affects both brain development and impairs brain function [42]. The results presented in this paper demonstrate that STC-1 cells express α and β nAChR subunits and their expression is modulated by nicotine. In addition, nicotine decreased the cellular content of BDNF in STC-1 cells which was dependent upon the presence of nAChRs. Consistent with the presence of nAChRs in STC-1 cells, α3 nAChR subunit expression was observed in intestinal enteroendocrine cells.

## Materials and Methods

### STC-1 cells

The mouse intestinal STC-1 cells were obtained from American Type Culture Collection (ATCC), Manassas, VA. STC-1 cells were cultured in Dulbecco’s Modified Eagle’s Medium (DMEM; Thermo Fisher Scientific, Waltham, MA) containing penicillin (100U/mL), streptomycin (100 μg/mL) and 10% fetal bovine serum (Life Technologies, Carlsbad, CA). Cells were maintained at 37°C in a humidified atmosphere of 5% CO_2_.

### RT-PCR

Total RNA from either STC-1 cells or mucosal cells obtained from the small intestine from C57/B6 mice was purified by using the TRIzol reagent (cat# 15596018, Thermo Fisher Scientific, MA, USA) and reverse transcripted by using High-Capacity cDNA Reverse Transcription Kit (cat# 4368814, Thermo Fisher Scientific, MA, USA). RT–PCRs for the detection of nAChR subunits and the other taste receptors were carried out by using MyTaq red mix (Bioline, Luckenwalde, Germany). Briefly, 2 μg total RNA was mixed with 2✕ Reverse Transcription Master Mix in a total volume of 20 μl per reaction. Reverse transcription were performed at 25°C for 10 min, then 37°C for 120 min, followed by 85°C for 5 sec and cooled to 4°C. Subsequently, 200 ng total cDNA was used as template, 35 to 40 cycles of PCR amplification were performed (initial denaturation at 95°C for 1 min, denaturation at 95°C for 15 sec, annealing for 15 sec at 53–60°C, and extension for 10 sec at 72°C). RT-PCR products were subjected to electrophoresis on a 1% agarose gel to determine the expression of nAChR subunits and other taste receptors. The mouse primers used to detect the presence of mRNAs for the nAChR subunits (chrna3, chrna4, chrna5, chrna6, chrna7, chrnb2, chrnb4), TRPM5, αENaC, TRPV1, GAPDH, BDNF and β-actin are shown in Table 1 and were synthesized by Thermo Fisher Scientific.

**Table 1:** Mouse primers for RT-PCR.

Gene product	NCBI Reference Sequence	Sequence	Amplified region
Chrna3 F	NM_145129.2	AGCTTAGCTGTGCTTCGGTG	98–469
Chrna3 R		ACTCCACCCCTTGGTAGTCA	
Chrna4 F	NM_015730.5	CTCGTCTAGAGCCCGTTCTG	79–500
Chrna4 R		GTCCGCGTTGTTGTAGAGGA	
Chrna5 F	NM_176844.4	TCTGACTGTCTTCCTGCTGG	1003–1368
Chrna5 R		CGGACGTCGTTCTCTTTCAC	
Chrna6 F	NM_021369.2	TCCGGTTTATGTCTGTGGCT	276–692
Chrna6 R		TGGGGTCCAGGTTATCACAC	
Chrna7 F	NM_007390.3	TGATTCCGTGCCCTTGATAG	911–1053
Chrna7 R		GAATGATCCTGGTCCACTTAGG	
Chrnb2 F	NM_009602.4	GACGGTGTACGCTTCATTGC	1508–1765
Chrnb2 R		GGTCACGGGATGAGTAGCTG	
Chrnb4 F	NM_148944.4	CTCTCTGTTCGCTCTGCTTCA	76–396
Chrnb4 R		AACACGATGTCAGGCAACCA	
TRPM5 F	NM_020277.2	CTTCCTGTTCACCTATGTCCTG	2322–2570
TRPM5 R		GAGGGCACCATTCTACAGGT	
αENaC F	NM_011324.2	ATGATGGTGGCTTCAACGTG	1713–1957
αENaC R		GAACTCTACACCCTTGGGCT	
TRPV1 F	NM_001001445.1	AGGGTGGATGAGGTGAACTG	2482–2678
TRPV1 R		TGGGTGCTATGCCTATCTCG	
T2R38 F	NM_001001451.1	ACTGAGCCACAACTACCAAG	261–393
T2R38 R		ATGGAGTGGAAAGGTGTGAG	
GAPDH F	GU214026.1	CTACCCCCAATGTGTCCGTC	772–1124
GAPDH R		TAGGGCCTCTCTTGCTCAGT	
BDNF F	NM_001048139.1	GGTATCCAAAGGCCAACTGA	1039–1221
BDNF R		CTTATGAATCGCCAGCCAAT	

F = forward; R = reverse

### Quantitative real-time PCR (qRT-PCR)

QRT-PCR was used to measure RNA transcripts of nAChR subunits, TRPM5 and PLCβ2. Total RNA was purified by using the TRIzol reagent (cat# 15596018, Thermo Fisher Scientific, MA, USA) and reverse transcripted using High-Capacity cDNA Reverse Transcription Kit (cat# 4368814, Thermo Fisher Scientific, MA, USA). Real-time PCR were conducted using carboxyfluorescein (FAM)-labeled probe sets from Integrated DNA Technologies (Coralville, IA): GAPDH: Mm.PT.39.1, Chrna3: Mm.PT.56a.43533732, Chrna7: Mm.PT.56a.8458406, chrnb4: Mm.PT.56a.11867247, Trpm5: Mm.PT.56a.12176814, plcβ2: Mm.PT.56a.17449722 (Invitrogen, Carlsbad, CA), Chrna4: Mm00516561_m1, Chrna5: Mm00616329_m1, Chrna6: Mm00517529_m1, Chrnb2:Mm00515323_m1. Results were calculated using the 2−ΔΔCt method based on GAPDH amplification, and normalized to the control group.

### Immunofluorescence

Immunofluorescence studies were performed on STC-1 cells and tissue sections of small intestine from C57/B6 mice and Sprague-Dawley rats. STC-1 cells were plated into 8-wells chamber slides (1X10^4^ cells/well) and fixed with ice-cold methanol for 10 min at -20°C. After washing with 1X PBS for 5 min and blocking with 3% goat serum for 1 h at room temperature, cells were stained with primary antibodies (1:50 dilution) in 1% goat serum at 4°C overnight. After washing, cells were incubated with fluorescent-conjugated secondary antibodies for 1 h. Nuclei were visualized with 1 μg/ml DAPI. Images were acquired with a 63X (1.4 numerical aperture) oil immersion objective and Zeiss LSM 700 confocal laser scanning microscope. Images were processed using Zen 2011 Image Processing Program, ImageJ (NIH software), and Photoshop CS2 software (Adobe Systems). A minimum of 90 cells were analyzed per staining. Primary antibodies against AChRα3 (sc-5590), AChRα4 (sc-5591), AChRα5 (sc-376979), AChRα7 (sc-1447), AChRβ2 (sc-11372), TRPM5 (sc-27366), T2R38 (sc-67109), β-actin (sc-47778), and BDNF (sc-12112) were obtained from Santa Cruz Biotechnology, CA, USA. The AChRβ4 antibody (cat# ab129276) was obtained from Abcam, MA, USA.

The animals were housed in the Virginia Commonwealth University (VCU) animal facility in accordance with institutional guidelines. All animal protocols were approved by the Institutional Animal Care and Use Committee (IACUC) of VCU. Two male C57/B6 mice (40–45 g) were used in this study. The animals were obtained from Charles River Laboratories, Wilmington, Massachusetts, USA. Animals were perfused with 4% paraformaldehyde/1×PBS for 5–10 min under anesthetic. The small intestine was excised and fixed in 4% paraformaldehyde/1×PBS for another 2 hours at 4°C, and dehydrated in 40% sucrose/1×PBS overnight at 4°C before embedding in O.C.T. Compound (Andwin Scientific, Cat 14-373-65). Sections (8 μm thick) were prepared using a CM3050S cryostat (Leica Microsystems) and applied on pre-coated microscope slides (Fisher Scientific, Cat 12-550-15). The sections were dried at room temperature for 20 min and immediately used for immunofluorescence. The procedure of immunofluorescence staining was same as described for STC-1 cells above. The co-localization of α and β nAChR subunits, and nAChRs with TRPM5 or T2R38 in STC-1 cells were evaluated using ImageJ software. Regions of Interest (ROIs) containing cell nuclei were monitored for immunofluorescence of the two probes.

### Immunoblotting and immunoprecipitation

Cells were washed with ice-cold PBS and lysed in modified radio-immunoprecipitation assay buffer (50 mM Tris-Cl (pH 7.4), 1% Nonidet P-40, 150 mM NaCl, 1 mM EDTA, 1 mM phenylmethylsulfonyl fluoride (PMSF), 1 μg/ml each of aprotinin and leupeptin, and 1 mM Na_3_VO_4_). For immunoprecipitation, 1 mg cell extract was incubated with 2 μg of antibody for 2 h at 4°C, followed by incubation with 40 μl Protein A/G-plus agarose (Santa Cruz Biotechnology) overnight at 4°C. The beads were washed with radio-immunoprecipitation assay buffer, and immune complexes were eluted by boiling in 2×SDS Laemmli loading buffer for 5 min. For immunoblotting, 20–50 μg protein was resolved by 10% SDS-PAGE and transferred to nitrocellulose membranes. Membranes were immunoblotted with primary antibodies, followed by HRP-conjugated secondary antibodies. Reactions were visualized by enhanced chemiluminescence reagents (Amersham Biosciences, Piscataway, NJ).

### Ca^2+^-imaging

STC-1 cells were grown on glass coverslips (Warner Instruments, Hamden, CT, USA) and washed three times with Ringer’s solution containing (in mM): 140 NaCl, 5 KCl, 1 CaCl_2_, 1 MgCl_2_, 10 glucose, 10 HEPES, pH 7.4. The cells were incubated with 16 μM Fura-2-AM (acetoxy methyl ester from Molecular Probes, Eugene, OR, USA) for 90–120 min at room temperature. Cells were washed with Ringer’s solution and coverslips were mounted in an experimental chamber (RC-26GLP, Warner Instruments; 0.7 ml volume) that fitted on to a Series 20 Chamber Platform (Warner Instruments). The cells were visualized through a water immersion 40X objective (Zeiss; 0.9 NA) with a Zeiss Axioskope 2 plus upright fluorescence microscope. The cells were imaged with a set-up consisting of a cooled CCD camera (Imago, Photonics) attached to an image intensifier (VS4-1845, VideoScope), an epifluorescent light source (Polychrome 5, Photonics), dichroic filter (415 nM), and 510 emission filter (40 nM band pass). The cells were alternately excited with 340 nM and 380 nM and the emitted light was imaged at 15s intervals. The temporal changes in fluorescence intensity ratio (F_340_/F_380_) in individual cells was analyzed using imaging software TILL Vision V3.3 (TILL Photonics, Martinsried, Germany). The changes in FIR were monitored under control conditions and after treating the cells with 50–250 μM nicotine or 5 mM denatonium in Ringer’s solution in the absence or presence of 10 μM Mec or dihydro-β-erythroidine (DHβE) (Sigma-Aldrich). In additional experiments, cells were first treated with 250 μM nicotine. Following this the cells were washed with control Ringer’s for 5 min. In the third step, the same cells were treated with 5 mM denatonium. The FIR values in each cell were normalized to 1 under control conditions.

### Measurement of BDNF by enzyme-linked immunosorbent assay (ELISA)

BDNF was measured in STC-1 lysates via a sandwich ELISA using the Promega Emax immune assay (Promega Corporation, Madison, WI, USA) according to the manufacturer’s protocol. ELISA plates were coated with anti-BDNF mouse antibody (mAb; 1:1000) and incubated overnight at 4°C. The next day the plate was washed and blocked with Blocking Buffer (Promega). BDNF standard or sample (100 μl) was added to each well and incubated for 2 h at room temperature. The plate was washed and anti-BDNF mAb (1:500; 100 μl) was added to each well and incubated for 2 h at room temperature. After washing, 100 μl of diluted anti-IgY HRP (horseradish peroxidase conjugate; 1:200) was added to each well and developed with TMB (3,3’,5,5’-tetramethylbenzidine) solution and 1 N HCl. The absorbance at 450 nm was measured using a VICTOR 2 plate reader and the concentration of BDNF in the samples was calculated from the standard curve and expressed as pg/mg protein [35]. BDNF assay was repeated 3 times. Each time the samples were run in triplicates containing 100 μl cell lysate from 1-2x10^6^ STC-1 cells/well.

### Data analysis

Student’s t test was employed to analyze the differences between the different data set.

## Results

### Localization of nAChRs in STC-1 cells

**Fig 1:**
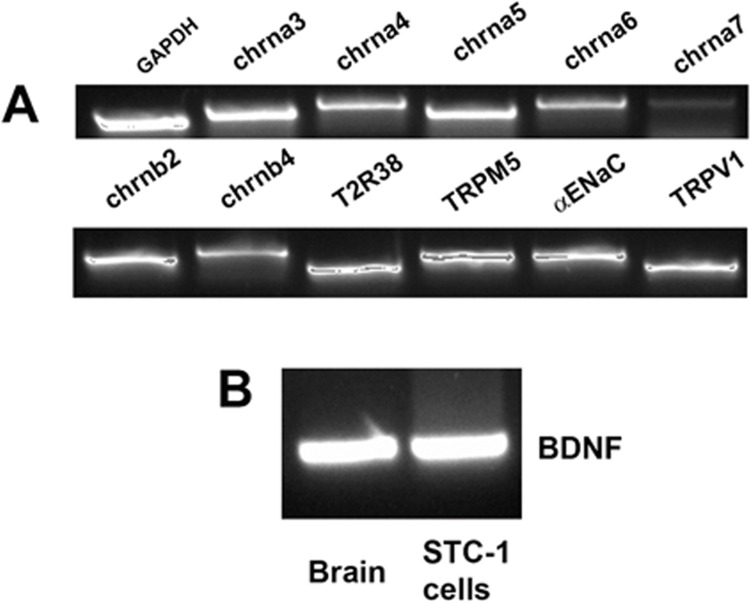
Expression of nAChR subunits, taste receptors, and BDNF in STC-1 cells. Consensus primers to amplify mouse nAChR subunits, taste receptor (T2R38), downstream signaling intermediate (TRPM5), and BDNF were designed based on the published sequences and are shown in Table 1. **(A)** Based on the predicted sizes of the PCR products (Table 1), we detected the mRNAs for the nAChR subunits: chrna3, chrna4, chrna5, chrna6, chrna7, chrnb2, and chrnb4 in the STC-1 cell cDNA sample. In addition, we detected the mRNAs for T2R38, TRPM5, α-ENaC, and TRPV1. **(B)** Based on the predicted size of the PCR product (Table 1), we also detected the mRNA for BDNF in STC-1 cells. Brain tissue was used as a positive control.

The mRNAs for chrna3, chrna4, chrna5, chrna6, chrna7, chrnb2, and chrnb4 nAChR subunits were detected from RNA isolated from STC-1 cells by RT-PCR (Fig 1A). In addition, we detected the mRNAs for T2R38 (a GPCR bitter taste receptor), TRPM5 (a cation channel necessary for the transduction of bitter, sweet, and umami taste), α-ENaC (a component of the amiloride- and Bz-sensitive salt taste receptor), and TRPV1 in STC-1 cells (Fig 1) [43].

**Fig 2:**
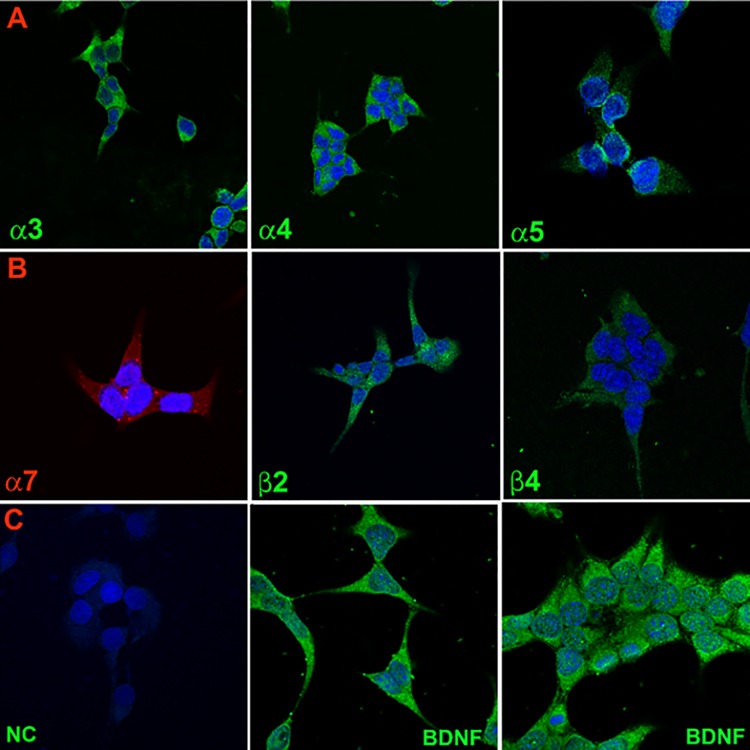
Immunofluorescence staining of nAChR subunits and BDNF in STC-1 cells. **(Panel A)** Immunostaining of nAChR α3, α4, and α5. **(Panel B)** Immunostaining of nAChR α7, β2, and β4. **(Panel C)** Negative control (NC) without primary antibody and immunostaining of BDNF. Blue color represents staining of cell nuclei with DAPI. The panels show merged confocal images of DAPI and secondary antibody fluorescence.

Immunohistochemical studies demonstrated specific staining of AChRα3, AChRα4, AChRα5 (Fig 2; Panel A), AChRα7, AChRβ2, and AChRβ4 nAChR subunits on STC-1 cells (Fig 2; Panel B). No staining was observed in STC-1 cells in the absence of the primary antibody (Fig 2; Panel C; negative control (NC)).

**Fig 3:**
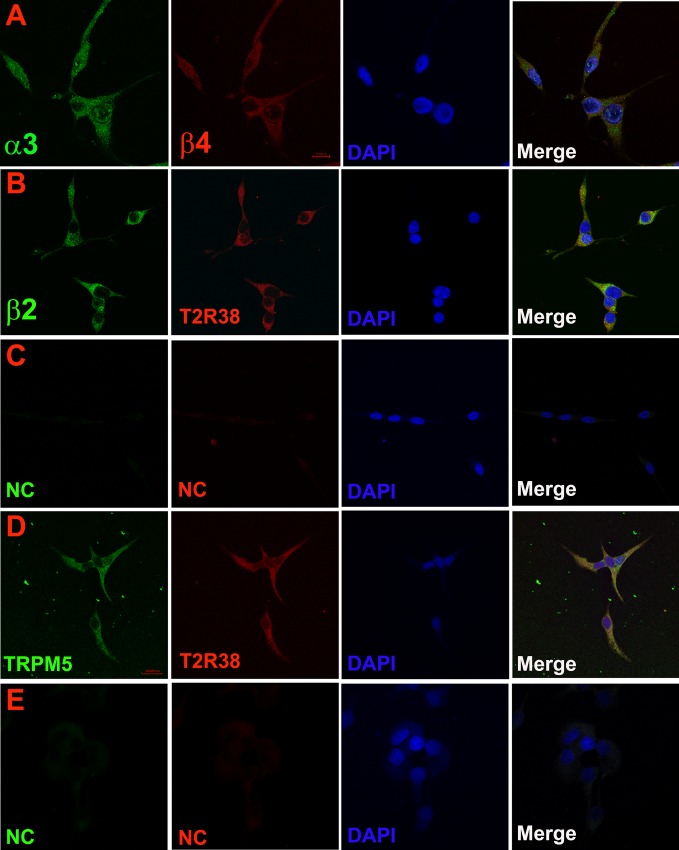
Co-localization of nAChR subunits with bitter taste receptors and downstream intracellular signaling intermediates in STC-1 cells. **(Panel A)** Immunostaining of AChRα3 and AChR**β**4. **(Panel B)** Immunostaining of AChR**β**2 and T2R38. **(Panel C)** Negative control (NC) without primary antibody. **(Panel D)** Immunostaining of TRPM5 and T2R38. **(Panel E)** Negative control (NC) without primary antibody. Blue color represents staining of cell nuclei with DAPI. The panels show merged confocal images of DAPI and fluorescence images of Donkey anti-Rabbit IgG (H+L) Secondary Antibody, Alexa Fluor® 488 conjugate (green) and Donkey anti-Rabbit IgG (H+L) Secondary Antibody, Alexa Fluor® 594 (red) conjugate.

Immunohistochemical studies using dual labelling with AChRα3 and AChRβ4 antibodies demonstrated co-localization of α3 and β4 nAChR subunits in the same STC-1 cells (Fig 3; Panel A). This suggests the presence of nAChRs composed of α3β4 subunits in STC-1 cells. Using dual labelling with AChRβ2 and T2R38 antibodies demonstrated that β2 nAChR subunit co-localizes with T2R38 in the same STC-1 cells (Fig 3; Panel B). Similarly, we observed that T2R38 and TRPM5 co-localize in the same STC-1 cells (Fig 3; Panel D). No staining was observed in STC-1 cells in the absence of the primary antibodies (Fig 3; Panels C and E). We used ImageJ software to evaluate co-localization of nAChR subunits with TRPM5 and T2R38. Ninety five to 97% of cells demonstrated co-localization of α3 and β4 nAChR subunits, T2R38 and β2 nAChR subunit, and TRPM5 and T2R38. Taken together, these results show that nAChRs are expressed in STC-1 cells that express bitter taste receptor T2R38 and TRPM5 ion channel.

**Fig 4:**
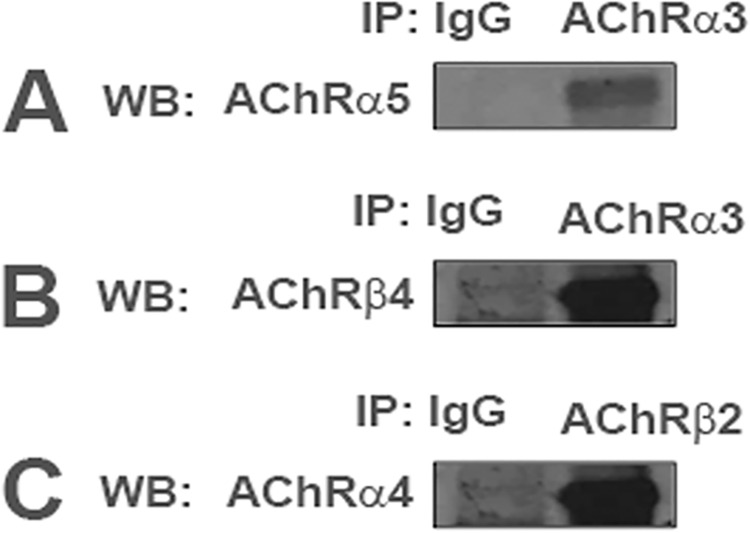
Co-IP of nAChRs in STC-1 cell lysates. In STC-1 cell lysates nAChRs α5 and β4 proteins were immunoprecipitated by AChRα3 antibody **(A, B)**, and nAChR α4 protein was immunoprecipitated by AChRβ2 antibody **(C).** IP = immunoprecipitation; WB = Western blot; IgG = negative control.

In STC-1 cell lysates, the α3 nAChR subunit was immunoprecipitated with AChRα5 antibody and AChRβ4 antibody (Fig 4A and 4B). In additional experiments, the β2 nAChR subunit immunoprecipitated with AChRα4 antibody (Fig 4C). These results suggest that in STC-1 cells nAChR subunits can assemble to form a simple α4β2 nAChR or complex nAChR(s) containing, in addition, α3 and α5 subunits. Thus, in addition to the classical taste receptors representing all of the five taste qualities, STC-1 cells express nAChR subunits.

### Effect of acute and chronic nicotine exposure on nAChR mRNA levels in STC-1 cells

**Fig 5:**
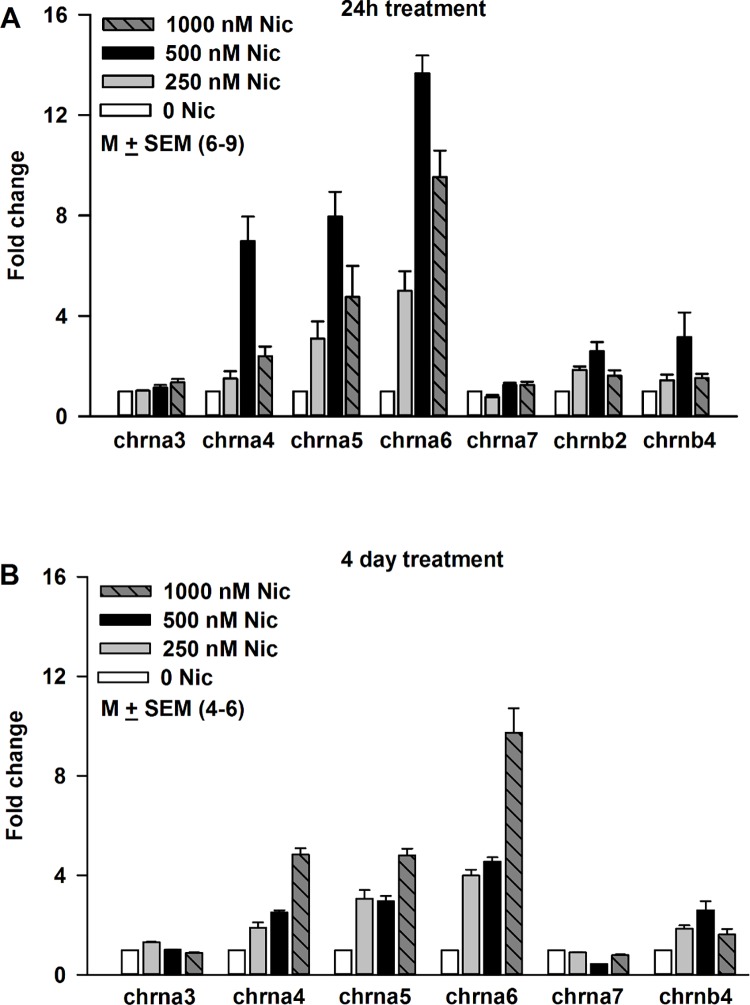
Effect of nicotine exposure on the nAChR mRNA expression level in STC-1 cells. **(A) After 24h treatment.** Relative to control at 250 nM nicotine the p values for chrna5, chrna6, chrna7, and chrnb2 were 0.0125, 0.0001, 0.0219, and 0.0001, respectively. Relative to control at 500 nM nicotine the p values for chrna4, chrna5, chrna6, chrna7, and chrnb2 were 0.0001, 0.0001, 0.0001, 0.0019, and 0.0012, respectively. Relative to control at 1000 nM nicotine the p values for chrna3, chrna4, chrna5, chrna6, chrnb2, and chrnb4 were 0.0272, 0.0041, 0.0125, 0.0001, 0.0184, and 0.0284. Relative to 250 nM nicotine at 500 nM nicotine the p values for chrna4, chrna5, chrna6, and chrna7 were 0.0003, 0.0002, 0.0001, 0.0006, respectively. Relative to 500 nM nicotine at 1000 nM nicotine the p values for chrna4, chrna5, chrna6, and chrnb2 were 0.0025, 0.01, 0.0146, and 0.042, respectively. **(B) After 4 day treatment**. Relative to control at 250 nM nicotine the p values for chrna3, chrna4, chrna5, chrna6, chrna7, and chrnb4 were 0.0001, 0.0022, 0.002, 0.0001, 0.0001, and 0.0001, respectively. After 4 day treatment, relative to control the p values for chrna4, chrna5, chrna6, chrna7, and chrnb4 at 500 nM nicotine were 0.0001, 0.0001, 0.0001, 0.0001, and 0.0001, respectively. Increasing nicotine concentration from 250 nM to 500 nM only produced a significant increase in mRNA level of chrna4 (p = 0.029). No significant changes were observed in the mRNA levels of the other nAChR subunits. In contrast, increasing nicotine from 500 nM to 1000 nM produced significant increases in all nAChR subunits investigated. After 4 day treatment, relative to 500 nM nicotine at 1000 nM nicotine the p values for chrna3, chrna4, chrna5, chrna6, chrna7, and chrnb4 were 0.0003, 0.0001, 0.0004, 0.0021, 0.0001, and 0.0001, respectively.

STC-1 cells were treated with 250 nM, 500 nM or 1000 nM nicotine for 24 h (Fig 5A). The mRNA levels of chrna4, chrna5 and chrna6 increased in a dose-dependent manner after nicotine treatment (Fig 5A). In STC-1 cells exposed to 1000 nM nicotine, the mRNA levels of chrna4, chrna5 and chrna6 decreased below the levels observed in cells treated for 24 h with 500 nM nicotine. This indicates that nicotine treatment for 24 h shows an inverted U shaped dose-response curve on the mRNA levels for chrna4, chrna5 and chrna6. While no significant changes in the mRNA levels of chrna3 and chrna7 were observed, chrnb2 and chrnb4 mRNA levels showed small increases at 500 nM nicotine (Fig 5A). Thus, acute nicotine exposure produces a differential increase in the mRNA levels of nAChRs.

Treating STC-1 cells with 250 nM, 500 nM and 1000 nM nicotine for 4 days, also produced increase in the mRNA levels of chrna4, chrna5, and chrna6 (Fig 5B). In contrast to the 24 h treatment, no significant increase in the mRNA levels of chrna4, chrna5, and chrna6 was observed when the nicotine concentration was increased from 250 nM to 500 nM nicotine. However, increasing nicotine to 1000 nM produced an increase in mRNA levels of chrna4, chrna5, and chrna6 nicotine that was greater than that observed with either 250 or 500 nM nicotine. Similar to the case with 24 h treatment, after 4 days of nicotine treatment no significant changes were observed in the mRNA levels of chrna3 and chrna7. However, a small increase in chrnb4 mRNA was observed at 500 nM nicotine (Fig 5B). These results show that acute and chronic exposure to nicotine produce different dose-response relationship on the nAChR mRNA levels in STC-1 cells.

### Effect of Mec on the nicotine-induced increase in nAChR mRNA levels in STC-1 cells

**Fig 6:**
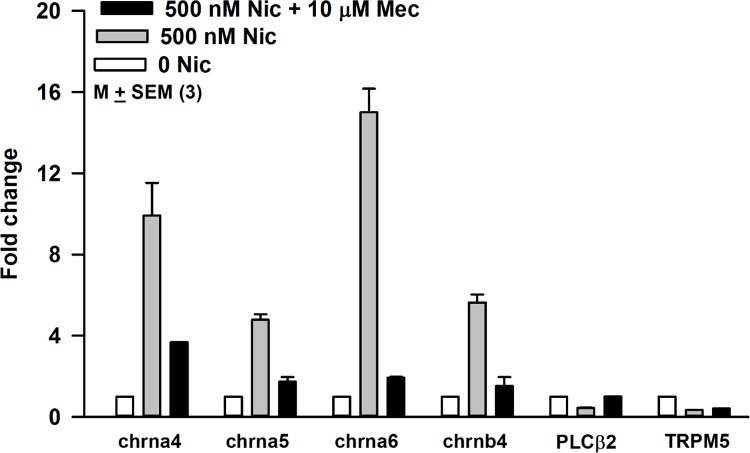
Effect of Mec on the nicotine induced increase in the nAChR mRNA expression level in STC-1 cells. STC-1 cells were treated with 0 Nic (control), 500 nM nicotine, 500 nM nicotine + 10 μM Mec or 10 μM Mec for 24h. In control STC-1 cells, the mRNA levels of chrna4 and chran6 increased as before. The increase in mRNA of chrna4 and chran6 by nicotine was blocked in the presence of Mec. Mec by itself did not affect the mRNA level of chrna4 and chran6 (data not shown).

Relative to STC-1 cells treated with 500 nM nicotine alone for 24 h, STC-1 cells exposed to 500 nM nicotine + 10 μM Mec, showed a significant decrease in the nicotine-induced increase in the mRNA levels of chrna4, chrna5, chrna6, and chrnb4 (Fig 6). These results suggest that the increase in chrna4, chrna5, chrna6, and chrnb4 mRNA levels in STC-1 cells depends upon the interaction of nicotine with the nAChRs.

Following exposure to 500 nM nicotine or 500 nM nicotine + 10 μM Mec for 24h, only small changes in mRNA levels of PLCβ2 and TRPM5 were observed (Fig 6). These results suggest that nicotine primarily affects the mRNA levels of chrna4, chrna5, chrna6, and chrnb4 but produces only minor effects on the mRNA levels of the intracellular signaling intermediates essential for the bitter, sweet, and umami taste transduction.

### Effect of nicotine exposure on nAChR protein levels in STC-1 cells

**Fig 7:**
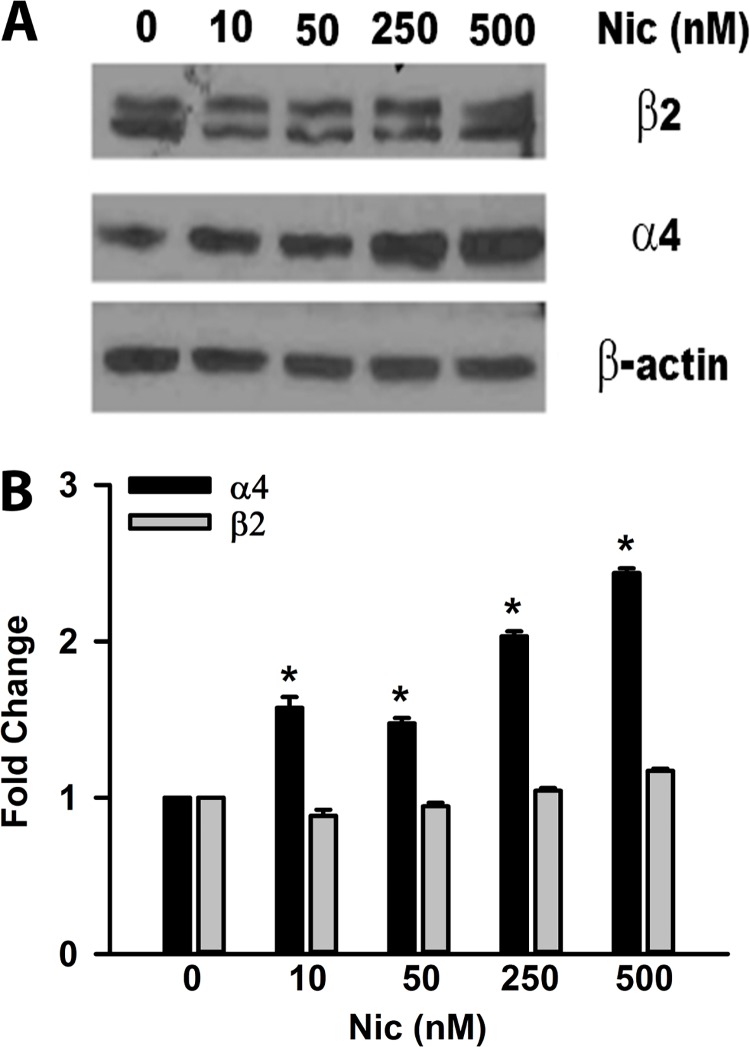
Effect of nicotine on the protein levels of α4 and β2 nAChR subunits in STC-1 cells. **(A)** STC-1 cells in culture were treated for 24h with varying concentrations of nicotine (0–500 nM). Samples containing 30 μg total protein were resolved by 10% SDS-PAGE, transferred to nitrocellulose membranes. Membranes were immune-blotted with primary antibodies against nAChRβ2, nAChRα4, and β-actin followed by HRP-conjugated secondary antibodies. Beta-actin was used as a protein loading control. Relative to control no significant changes were seen in β2 nAChR subunit protein level at nicotine concentrations tested. Relative to control, an increase in α4 nAChR subunit protein level was observed at 250 and 500 nM nicotine. The molecular weights of α4, β2, and β-actin are 75, 50 and 43 Kd. **(B)** Shows the ratio of the intensity of α4 and β2 bands relative to the intensity of the β-actin normalized to control (zero nicotine).

Exposing STC-1 cells to 250 nM or 500 nM nicotine for 24h also produced a dose-dependent increase in α4 nAChR protein expression (Fig 7A and 7B). However, no increase in β2 nAChR protein expression was observed at the nicotine concentrations used here (Fig 7A and 7B). It is important to note that exposing STC-1 cells to 500 nM nicotine also produced a significant increase in mRNA level of chrna4 but produced only minor changes in the mRNA level of chrnb2. These results show that for some of the nAChR subunits nicotine-induced increase in mRNA level is accompanied by an increase in protein expression.

### Effect of acute nicotine exposure on STC-1 cell Ca^2+^ ([Ca^2+^]_i_)

**Fig 8:**
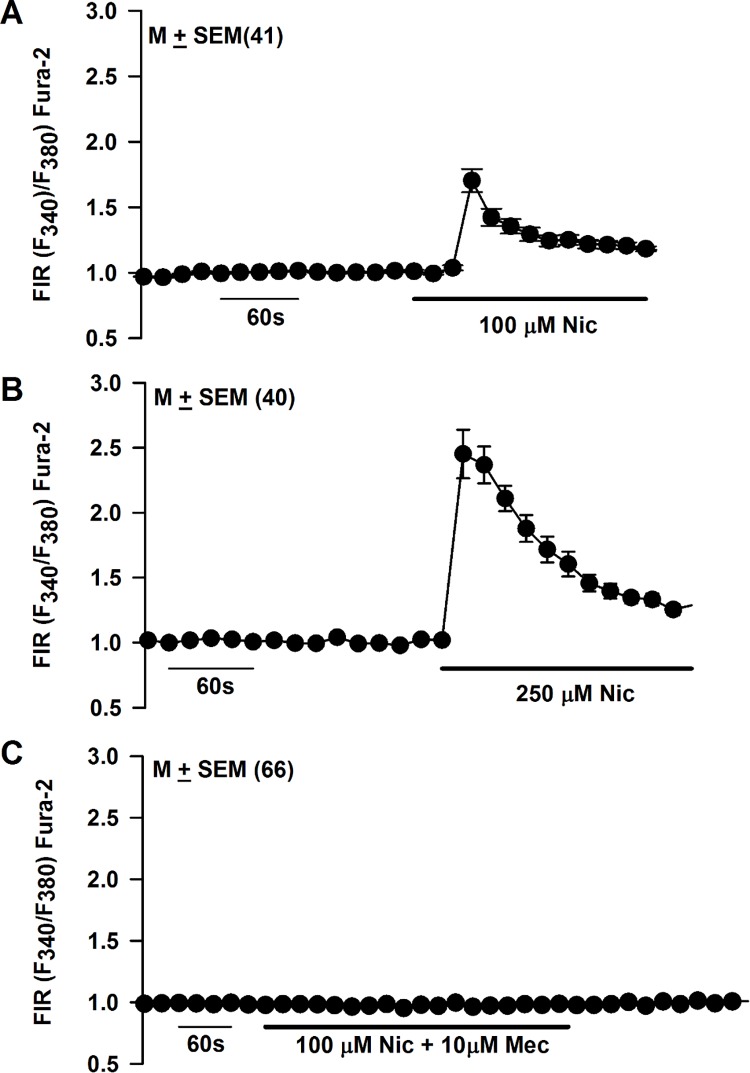
Effect of nicotine on STC-1 cell Ca^2+^. Changes in [Ca^2+^]_i_ were measured as temporal changes in FIR (F_340_/F_380_) in individual STC-1 cells loaded with Fura-2 as a response to 100 μM or 250 μM nicotine exposure. **(A)** In the representative experiment shown, 41 out of 112 cells (36.6% of the cells) responded with a rapid but transient increase in FIR when exposed to 100 μM nicotine. **(B)** In the representative experiment shown, 40 out of 66 cells (60.6% of the cells) responded with a rapid increase in FIR when exposed to 250 μM nicotine. Relative to 100 μM nicotine, at 250 μM nicotine, STC-1 cells responded with a significantly higher (p <0.0001, unpaired) mean maximum increase in FIR (1.70 ± 0.09 versus 2.45 ± 0.12). **(C)** Exposing STC-1 cells to 100 μM nicotine + 10 μM Mec produced no change in FIR in all 66 cells investigated. The values are presented as mean ± SEM of FIR of the number of cells (N).

STC-1 cell line represents a heterogeneous cell population [44]. Accordingly, we monitored changes in [Ca^2+^]_i_ as changes in FIR in individual STC-1 cells using Ca^2+^- imaging. In STC-1 cells loaded with Fura-2, 100 μM and 250 μM nicotine exposure produced a rapid but transient increase in FIR in a dose-dependent manner (Fig 8A and 8B). At 100 μM nicotine, 41 out of 112 STC-1 cells (36.6%) responded with a rapid increase in FIR. At 250 μM nicotine, 40 out of 66 STC-1 cells (60.6%) responded with a rapid increase in FIR (Fig 8B). At 250 μM nicotine, the nicotine-induced maximum increase in FIR varied between individual cells from 2.2 to 3.6. Relative to 100 μM nicotine, at 250 μM nicotine, STC-1 cells responded with a significantly higher (p <0.0084, unpaired) mean maximum increase in FIR (1.70 ± 0.09 versus 2.45 ± 0.14). Exposing STC-1 cells to 100 μM nicotine + 10 μM Mec produced no change in FIR in all 66 cells investigated (Fig 8C). In a separate set of 31 STC-1 cells, Mec alone produced no changes in FIR (data not shown). These results show that similar to TRCs [28, 29], STC-1 cells express functional nAChRs.

**Fig 9:**
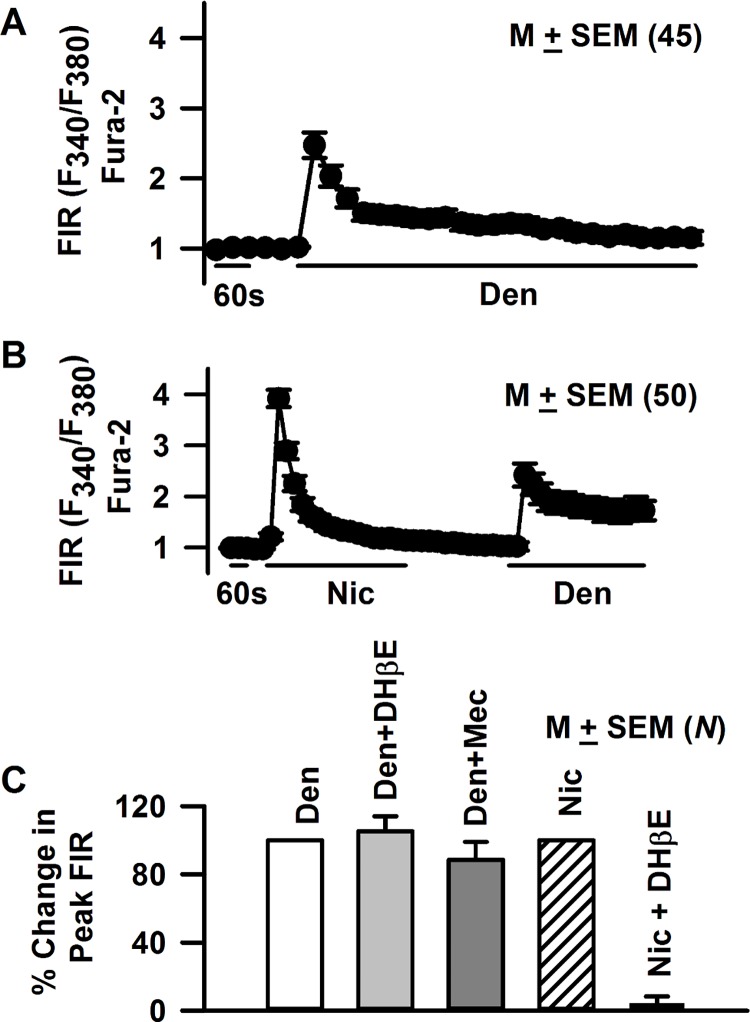
Effect of denatonium on STC-1 cell Ca^2+^. Changes in [Ca^2+^]_i_ were measured as temporal changes in FIR (F_340_/F_380_) in individual STC-1 cells loaded with Fura-2 as a response to 5 mM denatonium or 250 μM nicotine exposure. **(A)** Exposing STC-1 cells to 5 mM denatonium induced a transient increase in FIR in 45 out of 72 cells investigated (62.5% of the cells). **(B)** In separate experiments, cells were first treated with 250 μM nicotine (Nic). The cells were than washed with control Ringer’s solution for 5 min. After the wash the same STC-1 cells were treated with 5 mM denatonium. All 50 STC-1 cells that responded with an increase in FIR to 250 μM Nic also responded with an increase in FIR when treated with 5 mM denatonium. The values are presented as mean ± SEM of FIR of the number of cells (N). **(C)** STC-1 cells were first treated with 5 mM denatonium (Den) and then with Den + 10 μM DHβE or Den + 10 μM Mec. A different batch of STC-1 cells was first treated with 1 mM nicotine (Nic) and then with Nic + 10 μM DHβE. The values are presented as mean ± SEM of FIR of the number of cells (*N*). The peak change in FIR in the presence of Nic or Den in each cell was normalized to 100%. DHβE inhibited the nicotine-induced changes in FIR to near zero. Mec or DHβE produced no significant (p >0.05, unpaired) changes in FIR induced by Den. In each case FIR values were measured from 13–20 STC-1 cells.

Forty five out of 72 STC-1 cells (62.5% of cells) demonstrated a transient increase in FIR when exposed to 5 mM denatonium (Fig 9A). In separate experiments, STC-1 cells were first treated with 250 μM nicotine. The cells were then washed with control Ringer’s solution for 5 min. The same cells were then treated with 5 mM denatonium. Consistent with the results shown in Fig 8B, 50 out of 80 STC-1 cells (62% of the cells) responded with an increase in FIR when exposed to 250 μM nicotine (Fig 9B). The same 50 cells also demonstrated an increase in FIR after 5 mM denatonium treatment (Fig 9B). Similar results were obtained if STC-1 were first treated with denatonium and then with nicotine (data not shown). These results indicate that STC-1 cells that respond to nicotine also respond to denatonium [15]. Denatonium (5 mM) induced increase in FIR was not affected by Mec or DHβE (10 μM) (Fig 9C). In contrast DHβE (10 μM) inhibited the nicotine (1 mM) induced changes in FIR to near zero.

### Localization of BDNF in STC-1 cells

As shown in Fig 1B BDNF mRNA was detected from RNA isolated from STC-1 cells by RT-PCR. In addition, BDNF antibody showed immunostaining to STC-1 cells (Fig 2; Panel C; BDNF). These results show that BDNF is expressed in STC-1 cells. BDNF and its cognate receptor trkB are expressed mainly in type III TRCs [36, 37]. These results suggest that STC-1 cells are a good model to study taste receptor expression.

### Effect of nicotine on BDNF content and release in STC-1cells

**Fig 10:**
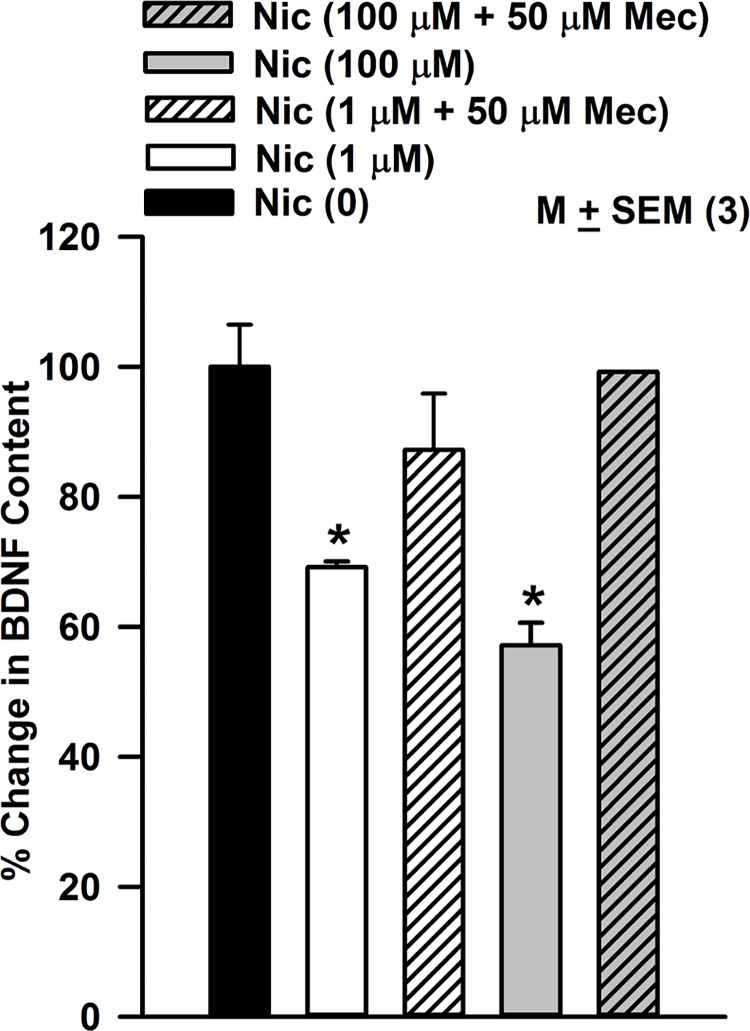
Effect of nicotine on STC-1 cell BDNF. STC-1 cells were treated with 1 or 100 μM nicotine (Nic) for 30 min in the absence and presence of 50 μM mecamylamine (Mec). BDNF was measured in cell lysates using ELISA. Nicotine decreased the BDNF content in STC-1 cells in a dose-dependent manner. The nicotine-induced decrease in BDNF was not observed in the presence of Mec. *BDNF value at 1 μM and 100 μM nicotine were significantly lower than the basal level (0 Nic) with p values of 0.0094 and 0.0044, respectively (paired). The BDNF values in the presence of Nic (1 or 100 μM) + 10 μM Mec were not different from the basal level (0 Nic) (p>0.05).

STC-1 cells were treated with 1 or 100 μM nicotine in the absence and presence of 50 μM Mec for 30 min. The BDNF cellular content under control conditions (pg BDNF/mg protein) normalized to 100% decreased with increasing nicotine concentration (Fig 10). The nicotine-induced decrease in BDNF was not observed in the presence of Mec. These results suggest that nicotine interacts with nAChRs and inhibits BDNF expression in STC-1 cells. Exposure of neonate rats to nicotine has been shown to cause a decrease in the expression of nerve growth factor and BDNF in hippocampus and frontal cortex [42].

### Expression of nAChRs in enteroendocrine cells of the gut

**Fig 11:**
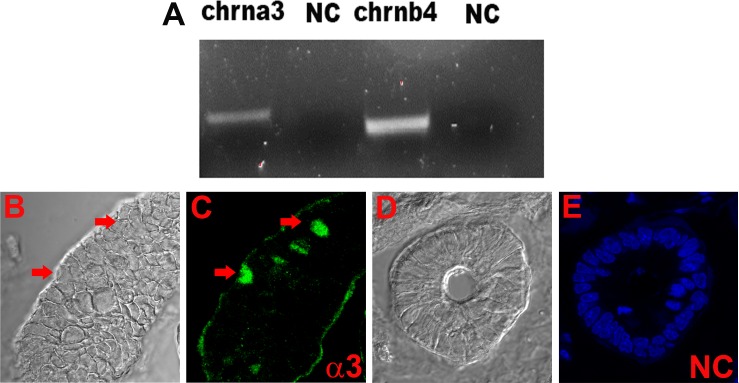
Localization of nAChRs in gut tissue. **(A)** Based on the predicted sizes of the PCR products (Table 1), we detected the mRNAs for chrna3 and chrnb4 in the intestinal mucosal cell cDNA sample. **(B and C)** Immunostaining of nAChR α3 in enteroendocrine cells in the gut (red arrows). We examined 3 different sections of the small intestine. In each of the 15 slides examined 2 or 3 enteroendocrine cells were positive for α3 nAChR in the crypts. **(D and C)** Negative control (NC) without primary antibody. Blue color represents staining of cell nuclei with DAPI. The panels C and E show merged confocal images of DAPI and secondary antibody fluorescence (Alexa Fluor® 488, green).

The mRNAs for chrna3 and chrnb4 were detected in the intestinal mucosal cell cDNA samples (Fig 11A). We examined α3 nAChR antibody binding in 3 different sections of the small intestine. In each of the 15 slides examined 2 or 3 enteroendocrine cells were positive for α3 nAChR in the crypts (Fig 11B and 11C). These results indicate that nAChRs are not only expressed in STC-1 cells but also in the enteroendocrine cells in the gut.

## Discussion

In addition to the known taste receptors and their downstream intracellular signaling intermediates: T2R38, PLCβ2, and TRPM5 (Figs 1–3 and 6) [14–16, 24, 25, 32], we detected the presence of mRNAs of α-ENaC and TRPV1 in STC-1 cells (Fig 1). Alpha-ENaC is a component of the amiloride- and benzamil sensitive ENaC, the sodium-specific salt taste receptor in fungiform TRCs [6–8]. The presence of α-ENaC in STC-1 cells is consistent with the observations that NaCl enhances [Ca^2+^]_i_ in these cells [45]. Although TRPV1 does not seem to be expressed in rodent TRCs [46, 47], it is expressed in STC-1 cells (Fig 1A). Capsaicin stimulated GLP-1 secretion from STC-1 cells in a Ca^2+^-dependent manner through TRPV1 activation [43].

### STC-1 cells express nAChRs

To date, eight α subunits (α2, α3, α4, α5, α6, α7, α9, and α10) and 3 β subunits (β2, β3, and β4) have been cloned from neuronal tissues. While α8 was identified in avian tissues, it has not been found in mammals [48, 49]. Neuronal nAChRs can be homopentamers or heteropentamers. Here, we present new results that show that STC-1 cells express nAChRs. The mRNAs of chrna3, chrna4, chrna5, chrna6, chrna7, chrnb2, and chrnb4 were detected in STC-1 cells using RT-PCR (Fig 1A). We also demonstrated the expression of α3, α4, α5, α7, β2, and β4 nAChR subunits in STC-1 cells using antibodies against these receptors (Figs 2 and 3). The observation that α3 and β4 nAChR subunits co-localize in the same cells (Fig 3; Panel A) indicates that these subunits combine to form functional nAChRs involving α3β4 combination in STC-1 cells. Our co-immunoprecipitation studies (Fig 4) further suggest that in STC-1 cells α and β subunits combine to form simple or complex receptors with multiple subunits [48, 49]. Consistent with the data shown here for STC-1 cells, immunoreactivity of α3, α4, α5, α7, and β2 nAChR subunits has previously been reported in cells in the lung expressing bitter taste receptors [50].

Although it is generally viewed that the vast majority of the antibodies against nAChR subunits are nonspecific [51, 52], in this study nAChR expression in STC-1 cells was confirmed by RT-PCR, qRT-PCR, immunohistochemical techniques, Western Blots, Ca^2+^-imaging and BDNF assay using ELISA. However, at present the various nAChR receptor subtypes expressed in STC-1 cells have not been identified. In central nervous system (CNS) α4β2* (* additional subunits may be present) and α7 nAChRs are the major heteromeric and homomeric receptors. However, more complex receptors combinations have been detected in different areas of the CNS [23].

### STC-1 cells express functional nAChR receptors

In previous studies [15, 33, 45] STC-1 cells responded with a rapid increase in [Ca^2+^]_i_ when exposed to stimuli representing all five taste qualities, including nicotine. Specifically, exposing STC-1 cells to denatonium at 0.1, 1, and 10 mM produced a dose-dependent increase in [Ca^2+^]_i_. At 1 mM, denatonium induced an increase in [Ca^2+^]_i_ in 33% of the STC-1 cell population. At 10 mM, denatonium induced a marked increase in [Ca^2+^]_i_ in 97% of the cells examined. In the same study, 10 mM nicotine was also reported to produce a rapid increase in [Ca^2+^]_i_ in 85% of the STC-1 cell population [15]. Our results demonstrate that significantly lower concentrations of nicotine (100 μM and 250 μM) can induce a rapid and dose-dependent increase in [Ca^2+^]_i_ in STC-1 cells. At 100 μM nicotine, only 36.6% of the STC-1 cells responded and at 250 μM nicotine 60.6% of the STC-1 cells responded with an increase in [Ca^2+^]_i_. The nicotine-induced increase in [Ca^2+^]_i_ was Mec- and DHβE-sensitive. This suggests that in STC-1 cells, at low nicotine concentrations, nicotine effects on [Ca^2+^]_i_ are due to its interactions with the nAChRs. Since DHβE is a competitive α4β2 nAChR antagonist, it suggests that one of the receptors through which nicotine exerts its effect on STC-1 cells is α4β2 nAChR [28].

Denatonium (5 mM) increased [Ca^2+^]_I_ in 62.5% of the STC-1 cells (Fig 9A). The cells which responded to nicotine (250 μM) also responded to denatonium (Fig 9B). In a previous study [15], at high concentrations (10 mM) nicotine and denatonium produced a marked increase in STC-1 Ca^2+^ in 85% and 97% of the cells, respectively. Denatonium induced increase in FIR was not affected by the presence of Mec or DHβE (Fig 9C). In our previous studies, Mec had no effect on the chorda tympani responses to quinine, NaCl or SC45647 [29]. These results suggest that the denatonium-induced changes in STC-1 cell Ca^2+^ are independent of nAChRs, but occur via its interactions with T2Rs. It is likely that at higher concentrations (e.g. 10 mM), nicotine produces its effects via its interactions with both nAChRs and T2Rs [15]. In the taste system, at 5 mM nicotine concentration the TRPM5-dependent and the TRPM5-independent components of the CT response accounted for approximately 50% of the total CT response [28]. Above 5 mM nicotine, the TRPM5-dependent (T2R component) fraction reached its maximum response and the TRPM5-independent response began to predominate. Consequently, at 10 mM nicotine, the T2R-TRPM5 component decreased to 41%, and at 20 mM nicotine it was 35% and further decreased to 30% in the high concentrations [28]. However, at present at 10 mM nicotine the contributions of the nAChR-dependent component and the T2R-TRPM5-dependent component to the nicotine induced increase in STC-1 [Ca^2+^]_i_ is not known [15].

STC-1 cells are heterogenous cell population [44]. At 250 μM nicotine, the maximum increase in FIR varied between individual cells from 2.2 to 3.6 (Fig 8B). This variation may arise due to the differences in α and β nAChR subunit expression, varying stoichiometry of α and β nAChR subunits or the presence of complex receptors with multiple α and β subunits [48, 49].

Denatonium induces secretion of GLP-1 from human enteroendocrine NCI-H716 cells [26]. Denatonium benzoate and HCl induced the release of CCK [25] in STC-1 cells. The effect of denatonium on the release of CCK in STC-1 cells was inhibited by chelating external Ca^2+^ with EGTA or in the presence of L-type voltage-sensitive Ca^2+^ channel blockers [33]. This suggested that bitter tastants increase [Ca^2+^]_i_ and CCK release through Ca^2+^ influx mediated by the opening of L-type voltage-gated Ca^2+^ channels (VSCCs) in STC-1 cells. However, at present, it is not known if nAChRs play a role in the nicotine-induced release of enteroendocrine peptides from STC-1 cells.

### Acute or chronic nicotine exposure upregulates nAChRs

Nicotine can desensitize as well as upregulate its receptors [53]. Our results show that upon exposure to 250–1000 nM nicotine for 24 h or 4 days produced a significant increase in the mRNA levels of chrna4, chrna5, and chrna6, with smaller changes in the chrnb4 mRNA (Fig 5). These changes in nAChR mRNA levels were inhibited when cells were treated with nicotine + 10 μM Mec (Fig 6). These results suggest that the increase in nAChR mRNA is dependent upon the interaction of nicotine with its receptor(s). The increase in mRNA for chrna4 was accompanied by an increase in α4 protein expression in STC-1 cells (Fig 7). Similar changes in nAChR mRNA and protein levels have been reported in other cells. Human dermal fibroblasts were treated with 10 μM nicotine in the absence and presence of 50 μM Mec for 24 h [54]. The relative nAChR subunit gene expression levels determined using subunit-specific RT-PCR primers demonstrated 1.8 to 3.8 fold increases in the expression of α3, α5, α7, β2, and β4 nAChR subunits. The presence of Mec abolished these changes [54]. Nicotine (100 nM) increased the levels of α7-nAChR mRNA and α7-nAChR transcription in human squamous cell lung cancer (SCC-L) cell lines and SCC-L tumors. The greatest expression of α7-nAChRs was observed from 100 nm to 10 μm at 96 h [55]. K-177, a stable cell line (HEK-293) expressing human α4 and β2 cells were exposed to 100 nM nicotine for 8 h. Nicotine caused upregulation of the α4β2 receptor [56]. Similar effects were reported at nicotine concentration of 1 μM [57]. When STC-1 cells were exposed to nanomolar concentrations of nicotine for 4 days, the mRNA levels for chrna4, chrna5, and chrna6 were also increased, but the dose-response relationship was different from the acute (24h) exposure (Fig 5). In male rats continually self-administering nicotine (approximately 1.5 mg free base/kg/day) was associated with upregulation of brain α4, α6, and β2 nAChR subunits [58]. Overexpressing nAChRs in mice increases sensitivity to nicotine [59].

Exposure to nicotine alters the trafficking and assembly of nAChRs, leading to their up-regulation on the plasma membrane [60]. Nicotine and its metabolite cotinine increased the number of α4β2 receptors on the plasma membrane and caused a redistribution of intracellular receptors. In contrast to this, cotinine exposure down-regulated α6β2β3 receptors. Cotinine and nicotine both alter the assembly of α4β2 receptors to favor the high sensitivity (α4)_2_(β2)_3_ stoichiometry [60]. Control subjects had a significantly greater density of immune-detectable mucosal epithelium α3 subunit, compared with ulcerative colitis patients [61]. Upregulation of α7 is systematically observed after incubation of lymphocytes with nicotine or α-bungarotoxin [62]. Taken together, nicotine exposure produces differential effects on the expression of nAChR mRNAs depending upon the dose, exposure time, and cell type [23]. Thus, nicotine exposure not only upregulates nAChRs in the CNS [58, 59] but also in peripheral tissues [54–56] and STC-1 cells.

### nAChR expression in gut tissue

Using *in situ* hybridization and immunocytochemistry α3 nAChR demonstrated widespread distribution in all the sections of human sigmoid colon examined, including mucosal epithelium, enteric ganglia, smooth muscle and follicular lymphoid [61]. In guinea pig small intestinal myenteric neurons maintained in culture antibodies selective for α3, α5, or β2 subunits stained most neurons, whereas a α7 subunit antibody stained very few neurons [63]. In this paper, we provide new evidence for nAChRs expression in the enteroendocrine cells of the gut (Fig 11). At present the subset of enteroendocrine cells that express nAChRs has not been characterized.

### Functional role of nAChR in STC-1 cells

At present the functional consequence of changes in differential upregulation of nAChRs in TRCs and enteroendoctine cells has not been investigated in detail. We hypothesize that differential upregulation of nAChRs in TRCs will alter the sensitivity to nicotine and ethanol [28] and in enteroendocrine cells the synthesis and release of peptide hormones. As shown in Fig 9, nicotine exposure reduced the BDNF content in STC-1 cells in a dose-dependent manner. This suggests that nicotine inhibits BDNF expression in STC-1 cells. BDNF is present in the gut and participates in survival and growth of enteric neurons, augmentation of enteric circuits, and stimulation of intestinal peristalsis and propulsion [34, 35]. In smokers a delay in gastric emptying was significantly correlated with increase in serum nicotine concentration [64]. Smoking abolished intense phasic contractions (phase III) activity in the stomach [65]. This indicates that nicotine elicits multiple physiological responses in the gut in animal models by acting at multiple sites [34, 35, 66].

Thus, our results represent an initial step in characterizing the functional role of nAChRs in enteroendocrine cells and TRCs. In future studies, it would be interesting to see if nicotine modulates the release of other enteroendocrine peptides from TRCs as well as enteroendocrine cells, including cell lines. It would also be interesting to investigate if TRCs in the taste buds also respond to changes in nAChR with acute or chronic nicotine exposure and alter taste responses to nicotine, acetylcholine and ethanol.
